# The color of a Dalmatian's spots: Linkage evidence to support the *TYRP1 *gene

**DOI:** 10.1186/1746-6148-1-1

**Published:** 2005-07-26

**Authors:** Edward J Cargill, Thomas R Famula, Robert D Schnabel, George M Strain, Keith E Murphy

**Affiliations:** 1 Department of Pathobiology, College of Veterinary Medicine, Texas A&M University, College Station, TX 77843 USA; 2 Department of Animal Science, University of California-Davis, Davis, CA 95616 USA; 3 Department of Animal Science, University of Missouri, Columbia, MO 65211 USA; 4 Department of Comparative Biomedical Sciences, School of Veterinary Medicine, Louisiana State University, Baton Rouge, LA 70803 USA

## Abstract

**Background:**

The distinctive coat pattern of a Dalmatian is the result of the interaction of several loci. While the encoded function of these genes is not fully understood, it is known the *Piebald*, *Ticking*, and *Flecking *loci interact to produce the Dalmatian's classic pigmented spots on a white background. The color of the pigmented spots in purebred Dalmatians can either be black or liver, but the locus responsible for color determination is unknown. Studies have been conducted to determine the underlying genes involved in coat color determination in the dog, e.g., in the Labrador Retriever, but none to date have addressed black versus liver in the Dalmatian.

**Results:**

A genome scan was conducted in a multi-generational kindred of Dalmatians segregating black and liver spot color. Linkage analysis was performed using a total of 113 polymorphic microsatellite markers from the kindred. Linkage was found between spot color and a single microsatellite marker, FH2319 (LOD = 12.5) on chromosome 11.

**Conclusion:**

The *TYRP1 *(*Brown*) locus is located at position 50.1 Mb on chromosome 11, which is approximately 0.4 Mb from marker FH2319. Given the recent characterization of *TYRP1 *genetic variations in the dog and the linkage evidence reported here, *TYRP1 *is likely responsible for the spot color variation of black versus liver seen in the Dalmatian.

## Background

The distinctive coat pattern of a Dalmatian is the result of the interaction of several genes. Specifically, it is known the *extreme piebald *allele of the *Piebald *locus, in conjunction with the *ticked *allele of the *Ticking *locus, and *nonflecked *allele of the *Flecking *locus, produces pigmented spots on a white background [1]. The color of the pigmented spots in registered Dalmatians is black or liver [2], but the locus responsible for the color variation has not been identified in the Dalmatian. Other colors are rare, but can be found [3].

Several classic pigmentation loci, such as *Agouti*, *Extension*, and *Brown*, have been characterized at the molecular level. The *Brown *locus describes tyrosinase related protein 1 (Tyrp1), which controls the production of eumelanin in melanocytes [4]. In the dog, the dominant *wild-type *allele results in black eumelanin while the recessive *brown *allele results in brown eumelanin [1]. The *Extension *locus has been found to be the melanocortin 1 receptor (Mc1r) protein [5], which has an epistatic relationship with the *Agouti *locus [1]. The epistatic relationship between *Agouti *and *Extension *acts as a switch for melanocytes to produce either phaeomelanin or eumelanin, with certain alleles resulting in a red or reddish-brown color [6].

All Dalmatians are homozygous for the *Piebald*, *Ticking*, and *Flecking *loci (or they would not display the classic spotting pattern) complicating characterization of these loci through standard techniques, such as linkage analysis, since there is not a segregating phenotype to detect. However, black and liver spot color is a detectable phenotype that segregates in Mendelian fashion. The objective of this study was to utilize a previously described multi-generational kindred of Dalmatians [7] to conduct a genome scan and perform linkage analysis to detect linkage between spot color and a microsatellite marker. Detection of linkage will provide insight as to which locus may be responsible for black versus liver in the Dalmatian.

## Results

**Figure 1:**
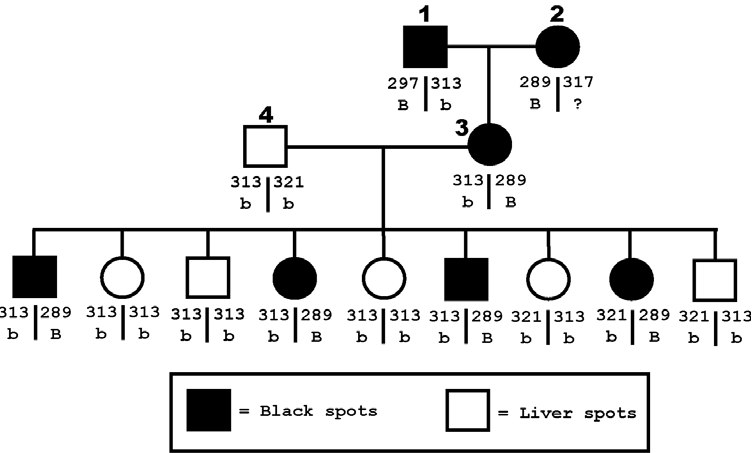
**Subset family of Dalmatians segregating black and liver spots. **Marker FH2319 genotypes (allele sizes ranging from 289 bp to 321 bp) and *Brown *locus alleles (*B *= wild type; *b *= brown) for a subset family of Dalmatians segregating black and liver spot colors.

Twopoint linkage analysis for spot color was performed using SOLAR v2.1.4 [14] and 113 polymorphic markers from the Dalmatian kindred. Marker FH2319 resulted in a statistically significant LOD score of 12.5 [8,9]. FH2319 was previously mapped to chromosome 11 at position 49.7 Mb [10]. None of the remaining 112 markers resulted in a LOD score above the standard significance threshold of 3, including four additional polymorphic markers on CFA11. The spot color and corresponding genotypes for a subset of the kindred are shown in Figure 1.

## Discussion

A genome scan was performed on a multi-generational kindred of Dalmatians, previously analyzed for heritability and segregation of deafness [7]. Spot color has been shown to not be associated with deafness in the Dalmatian [7]. Twopoint linkage analysis using SOLAR produced a LOD score of 12.5 for marker FH2319 on chromosome 11. Based on the canine genome map [10], the *TYRP1 *(*Brown*) locus is at position 50.1 Mb, which is approximately 0.4 Mb from FH2319. Given the recent characterization of *TYRP1 *genetic variations in the dog [11] and the linkage evidence reported here, *TYRP1 *is likely responsible for the spot color variation of black versus liver seen in the Dalmatian.

Included in Figure 1 are suspected alleles of the *Brown *locus for the depicted dogs. Dog 3 is heterozygous for the dominant *B *allele of the *Brown *locus (producing black spots) while dog 4 is homozygous for the recessive *b *allele of the *Brown *locus (producing liver spots). Progeny of dogs 3 and 4 reveal marker FH2319 allele 289, in this family, is linked with the *B *allele of the *Brown *locus. Additional alleles of marker FH2319 were also linked with the *B *allele in the Dalmatian kindred (data not shown). Due to this fact, linkage alone is not enough to establish if a dog with an unknown *Brown *locus genotype (such as dog 2 in Figure 1) is homozygous or heterozygous for the dominant allele.

A recent study [11] examined genetic variations in the *TYRP1 *and *MC1R *loci in numerous dog breeds to determine the effect of the variations on black and brown pigmentation. While this work characterized the associations of specific mutations found in these genes with the pigmentation phenotypes examined, only one Dalmatian (with black spots) was included. Based on examination of the kindred reported here, the *TYRP1 *gene should be further characterized in the Dalmatian to determine the specific genetic variations associated with the dominant and recessive alleles of the *Brown *locus.

## Conclusion

Statistically significant linkage was found between marker FH2319 and spot color in a kindred of Dalmatians. FH2319 is 0.4 Mb from the *TYRP1 *locus, supporting evidence that its gene product is responsible for the black or liver color of a Dalmatian's spots. Further characterization of *TYRP1 *in the Dalmatian is warranted to determine the specific genetic variations causative for color variation.

## Methods

### Spot color

Spot color was recorded from the Dalmatian kindred as previously described [7]. There were a total of 139 dogs with black spots (61 males, 78 females) and 60 with liver spots (26 males, 34 females). For the purpose of linkage analysis, spot color was coded as a binary trait with '0' for black and '1' for liver.

### DNA samples

Genomic DNA was isolated from 117 dogs (54 males, 63 females) of the Dalmatian kindred reported [7]. The remaining 149 dogs of the kindred were unavailable for collection of a DNA sample. The PUREGENE DNA Isolation Kit (Gentra Systems, Minneapolis, MN, USA) was used to extract genomic DNA from either whole blood or buccal swabs according to the manufacturer's specifications.

### Microsatellite markers

Microsatellite markers were amplified and resolved in multiplexed sets as described [12]. A linkage map of the markers based on the Dalmatian kindred was previously described [13]. Out of 172 total markers analyzed, 113 were polymorphic in the assembled Dalmatian kindred.

### Linkage analysis

Twopoint linkage analysis was performed using the program SOLAR v2.1.4 [14] according to the developers' instructions.

## Authors' contributions

EJC participated in design of the study, collected samples, performed the genome scan and linkage analysis, and drafted the manuscript. TRF was involved with the linkage analysis and review of the manuscript. RDS was involved with the linkage analysis and review of the manuscript. GMS was involved with collection of samples and review of the manuscript. KEM conceived of the study, participated in its design and coordination, and review of the manuscript.
